# The effects of zinc oxide nanoparticles on the oxidative stress, caspase-3, cytokine and immunity in rats

**DOI:** 10.17221/11/2025-VETMED

**Published:** 2025-10-30

**Authors:** Meryem Gultekin, Meryem Eren, Fatih Dogan Koca, Caglar Kaan Bozbek, Nermin Develi

**Affiliations:** ^1^Department of Biochemistry, Faculty of Veterinary Medicine, Erciyes University, Kayseri, Turkiye; ^2^Department of Aquatic Animal and Diseases, Faculty of Veterinary Medicine, Erciyes University, Kayseri, Turkiye; ^3^Graduate School of Health Science, Erciyes University, Kayseri, Turkiye

**Keywords:** caspase-3, cytokine, immunoglobulin, oxidative stress, zinc oxide nanoparticles

## Abstract

This study was conducted to examine the effects of zinc oxide nanoparticles (ZnO NPs) on the malondialdehyde (MDA) concentrations, superoxidase dismutase (SOD), glutathione peroxidase (GPx) and caspase-3 (CASP3) activities, tumour necrosis factor-alpha (TNF-α) and interleukin-6 (IL-6), immunoglobulin (Ig) E, G, M and zinc (Zn) concentrations in the serum/plasma and liver tissues of rats. Forty Wistar Albino rats were separated into five equal groups as the control, 5 and 10 mg/kg, b.w./day ZnO, 5 and 10 mg/kg b.w./day ZnO NPs were administered i.p. every other day for 14 days. The plasma MDA and plasma/liver TNF-α concentrations increased in the 10 mg/kg ZnO, 5 and 10 mg/kg ZnO NPs groups. The plasma SOD, CASP3, plasma/liver GPx activities and serum Zn concentrations increased in all the Zn groups. The highest SOD, GPx and CASP3 activities were detected in the 5 mg/kg ZnO NPs group. The plasma IgG concentrations increased, especially in the ZnO NPs groups. The study findings suggest that 5 mg/kg ZnO NPs could potentially have an ameliorative effect on the possible adverse effects of oxidative stress. These nanoparticles demonstrate their ability to combat oxidative stress by increasing the plasma/tissue SOD, GPx, and CASP3 activities, TNF-α, and IgG concentrations. However, the effectiveness of the nanoparticles may vary depending on the synthesis method, application time, and concentration.

Nanoscience is a branch of science that generally focuses on the synthesis, application, and characterisation of materials at the nanoscale (Vijayaraghavan and Ashokkumar 2017; Behera et al. 2020). Nanoparticles are preferred in many fields such as biotechnology, biology, medicine, and veterinary medicine due to their small size (1–100 nm) and remarkable ability to interact with biomolecules on cell surfaces and in intracellular environments (Troncarelli et al. 2013; Vijayaraghavan and Ashokkumar 2017; Khan et al. 2019; Tang et al. 2016). In veterinary medicine, nanotechnology is seen to have many application areas, including animal health, production and nutrition, as well as tissue engineering, diagnostic tools, vaccine production, and modern disinfectants (Troncarelli et al. 2013; Masroor et al. 2022; Figure 1).

**Figure 1:**
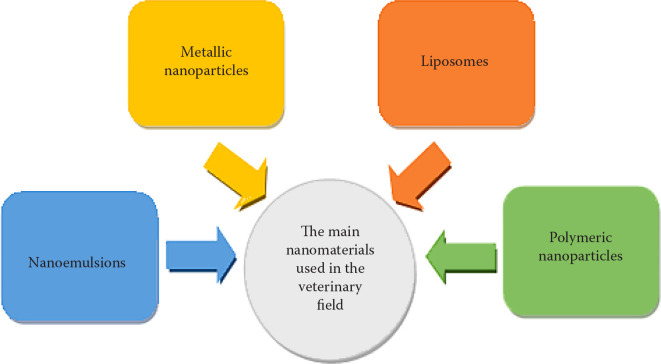
The main nanomaterials used in the veterinary field (Altav et al. 2019)

When taken into the body, nanoparticles (NPs) can remain in the circulatory system for a long time and reach different organs and body parts thanks to their small size and surface modifications.

As these particles can pass through the small intestine and reach the blood, lungs, brain, heart, spleen, kidneys, liver, stomach, and intestines (Fard et al. 2015; Salim et al. 2024), exposure to nanoparticles is increasing through various routes such as inhalation, dermal contact, and the gastrointestinal tract (Bayat et al. 2023; Farokhcheh et al. 2021; Salim et al. 2024).

The biological properties of nanomaterials, which are widely used due to their large surface areas, increased reactivity, high chemical stability and superior potential properties (Wahab et al. 2013; Moatamed et al. 2019), are affected by various factors such as the chemical composition, shape, surface charge and size (Card et al. 2011; Pietroiusti et al. 2017). Chemical, biological and physical methods are generally used in the synthesis of nanoparticles. Metallic nanoparticles, such as gold (Au), silver (Ag), copper (Cu), iron (Fe) and zinc oxide (ZnO), can be synthesised using various methods, and their characterisation is crucial for understanding their properties and potential applications (Wahab et. al. 2013; Idris and Roy 2023). The synthesis of nanoparticles by green synthesis enables the production of biogenic NPs that are compatible for biomedical and other applications. These methods particularly involve the use of plant extracts. NPs synthesised by the green synthesis method are environmentally friendly and can be synthesised easily, in a cost-effective manner, and in a way that reduces the risk of toxicity. Therefore, biomolecules found in plant extracts, such as alkaloids, terpenoids and phenolic compounds, can be used to reduce metal ions to nanoparticles in an environmentally safe, harmless, cost-effective, easily scalable, and one-step green synthesis process. At room temperature and pressure, the biogenic reduction of metal ions to the main metal can be carried out very quickly and easily (Mandal et al. 2022; Ali 2023). Moreover, it also helps in understanding the properties of metallic nanoparticles, such as the shape, size, structure, surface interactions, crystallinity, and chemical composition, during their characterisation (Mandal et al. 2022; Idris and Roy 2023).

Zinc is a cofactor of many enzymes and an important component of the antioxidant enzyme superoxide dismutase (SOD); It is an important element involved in processes such as the antioxidant defence system, immune response, protein and DNA synthesis, growth and development, thyroid metabolism, nerve conduction and wound healing (Zhao et al. 2014; Maaress and Hasse 2016). In veterinary medicine, there are studies reporting the beneficial effects of inorganic and organic forms of zinc on both metabolic and performance indicators (Wang et al. 2016; Sizova et al. 2019; Abdel-Magied and Shedid 2020).

Although the widespread use of ZnO NPs in commercial products and their release into the environment increases the possibility of exposure in the organism, the lack of interest in toxicity assessments (Ansar et al. 2018) has raised concerns about the potential toxicity of nanoparticles (Aboulhoda et al. 2020; Bayat et al. 2023). So far, there is limited information available regarding the effectiveness of nanoparticle forms of various minerals, including Zn (Ahmadi et al. 2013; Behera et al. 2020; Majd et al. 2021a; Majd et al. 2021b; Salim et al. 2024). Zinc oxide nanoparticles (ZnO NPs) are the most researched and widely used nanostructured materials among different metal oxides such as gold, silver and iron (Wahab et al. 2013; Faizan et al. 2020). This is due to their multifunctional physical and chemical properties and simple synthesis methods (Wahab et al. 2013; Mandal et al. 2022). One of the nanominerals commercially used to improve the growth rate, immunity, and reproductive status of livestock and poultry is ZnO NPs (Abdel-Wareth et al. 2022; Mozhiarasi et al. 2024). Additionally, ZnO NPs possess properties such as high UV light absorption, optical, antimicrobial, dermatological, digestibility, high catalytic efficiency, strong adsorption ability, high surface activity, and bioavailability (Xiaoli et al. 2017; Heidai-Moghadam et al. 2019; Mandal et al. 2022). Therefore, it is widely used in different industrial and commercial fields such as medicine, biomedical applications, antibacterial and cancer treatment drugs, cosmetics and personal care products, agriculture, food products, paints, and the electronics industry (Xiaoli et al. 2017; Heidai-Moghadam et al. 2019; Moatamed et al. 2019; Aboulhoda et al. 2020; Bayat et al. 2023; Farokhcheh et al. 2021; Khayal et al. 2021; Mandal et al. 2022).

Recently, the use of ZnO NPs has become controversial. Although ZnO NPs easily pass through the cell membrane and provide therapeutic benefits by reacting with cellular macromolecules, they have been determined to cause cytotoxic effects in different organs and cause oxidative stress (Khayal et al. 2021). Therefore, it is crucial to determine both the protective effects and the toxicological effects of ZnO NPs. Since there are not enough studies (Ahmadi et al. 2013; Behera et al. 2020; Majd et al. 2021a; Majd et al. 2021b; Salim et al. 2024) on the effect of ZnO NP, in this study, the effects of ZnO NP and the bulk ZnO on the malondialdehyde (MDA) concentrations, super oxidase dismutase (SOD), glutathione peroxidase (GPx) and caspase-3 (CASP3) activities, tumour necrosis factor-alpha (TNF-α) and interleukin-6 (IL-6), immunoglobulin (Ig) E, G, M and zinc (Zn) concentrations in the serum/plasma and liver tissues in rats were investigated.

## MATERIAL AND METHODS

### Green synthesis and characterisation of ZnO NPs

For the biosynthesis of ZnO NPs, an extract of *Lavandula officinalis* was utilised as both a capping agent and for reducing. *Lavandula officinalis*, purchased from herbalists, was washed and dried (70 °C) in the laboratory. Classically, 10 g of powdered *L.* *officinalis* was kept at 90 °C in 100 millilitres of distilled water. The resulting solution was subsequently filtered using Whatman No. 1 filter paper, and the extract was kept at +4 °C for use in synthesis studies. The plant extract (42.5 ml) was stirred with zinc nitrate (2 g) (about one hour at 60 °C). The solution was calcinated at 400 °C (Doan Thi et al. 2020). The characteristic absorption peak, surface charge, morphology, organic compounds, and crystallinity of ZnO NPs were detailed by ultraviolet (UV) Vis Spectrophotometer (UV-Vis), the Zeta potential, Scanning Electron Microscopy (SEM), X-ray Powder Diffraction (XRD), and Fourier Transform Infrared Spectroscopy (FT-IR). The ZnO bulk purchased from Merck (Cat. No. 108849.0500) was used for comparison.

### Animal material and experimental design

For this study, the ethics committee’s decision 21/27, dated February 3, 2022, was obtained from Erciyes University Animal Experiments. In the study, 40 male Wistar Albino rats (200–250 g) were divided into five experimental groups, each consisting of eight rats.

Group 1 was designated the control group and received 0.5 ml of physiological saline. Group 2 was administered 5 mg/kg of body weight (b.w.) per day of zinc oxide (ZnO), while Group 3 received 10 mg/kg (b.w.) per day of ZnO. Group 4 was given 5 mg/kg (b.w.) per day of ZnO NPs, and Group 5 received 10 mg/kg (b.w.) per day of ZnO NPs.

All the applications were made through the intraperitoneal (i.p.) route once every two days for 14 days (Moatamed et al. 2019; Shkal et al. 2020). The animals were fed *ad libitum* with a ration that would meet their daily nutritional needs.

The doses of ZnO NPs were established based on the no observed adverse effect level (NOAEL), specifically citing a dose of 50 mg/kg of ZnO NPs found to be effective, as reported by Rani et al. (2018) in their study involving the intraperitoneal administration of ZnO NPs to rats.

Four rats were placed into each cage sized 36 × 24 × 19 cm available in the research units of Erciyes University, Faculty of Medicine, Hakan Çetinsaya Experimental and Clinical Research Center (DEKAM). The animals were kept for five days without any application in order to adapt to the environment. The ambient temperature was kept at 15–20 °C. Laboratory conditions were controlled throughout the experiments to maintain a cycle (12-hour light/12-hour dark). Procedures were performed under anaesthesia to ensure the animals did not experience pain during the experiments.

### Sample collection

On the 14^th^ day of the experimental study, the rats were anaesthetised after 12 h of fasting. Blood samples were taken from their hearts and placed into tubes without anticoagulants for the serum separation and tubes containing Ethylenediaminetetraacetic acid (EDTA) for the plasma analysis. After centrifugation at 1 300 × *g* for 10 min at 4 °C, the serum and plasma were separated. The liver tissue samples were collected and kept at –80 °C until the analyses were performed. The levels of the MDA, SOD, GPx, CASP3 activities, TNF-α, IL-6, IgE, IgG and IgM were measured in both the plasma and liver samples, while the zinc (Zn) levels were assessed in the serum samples.

### Preparation of the liver tissue homogenates

The liver tissue taken for biochemical analyses was rinsed with distilled water to remove any blood and other residual substances, washed with cold 0.9% NaCl and dried with blotting paper. Then they were wrapped in aluminium foil and stored at 80 °C. Before the analysis, the liver tissue was weighed approximately 0.1 g on a precision balance, and 0.9 millilitres of 1 : 10 diluted phosphate buffer was added and ground with a glass homogeniser. The homogenate was transferred into tubes and centrifuged at 3 100 *g* (Hettich Zentrifugen, Universal 320 R, Germany) at +4 °C for 5 minutes. The supernatants were separated and kept at –80 °C until further analysis of the MDA, SOD, GPx, CASP3, TNF-α, IL-6, IgE, G and M (Panda et al. 2012).

### Biochemical analyses

The plasma and tissue MDA levels, SOD, GPx and CASP3 activities, TNF-α, IL-6, IgE, IgG and IgM concentrations were measured using an enzyme-linked immunosorbent assay (ELISA) (ELx50; Bio-Tek, Winooski, USA) with commercially available kits (Cat. Nos. 201-11-0157; 201-11-0169; 201-11-1705; 201-11-5114; 201-11-0765; 201-11-0136; 201-11-0453; 201-11-0454; 201-11-0455; Sunred-Bio, Shanghai, P.R. China, respectively). The serum Zn concentrations were assessed using an ICP/MS (Agilent 7500a series).

### Statistical analysis

Statistical analyses were performed using SPSS v20.0 for Microsoft (IBM Corporation, USA). Differences between the groups were assessed with a one-way analysis of variance (ANOVA). When a significant *F*-score was obtained, Duncan’s multiple range test was applied to reveal the differences between the groups. All the data are presented as means ± standard error of the mean (SEM), and value of *P* < 0.05 indicates that the difference between the experiment groups is significant.

## RESULTS

### Characterisation of the ZnO NPs

**Figure 2:**
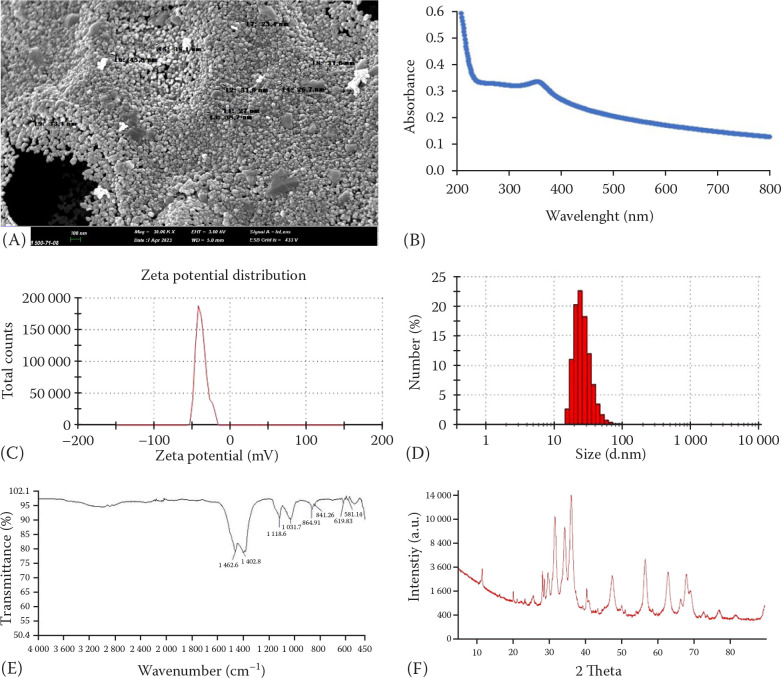
SEM images (A), UV-Vis measurement (B), Zeta potential (C), DLS analysis (D), FT-IR diagram (E), XRD diagram (F) of the ZnO NPs

The SEM images demonstrated that ZnO NPs tend to be spherical and agglomerate at an average size of 33 nm (Figure 2A). In this study, the characteristic absorbance of ZnO NPs was recorded at 364 nm (Figure 2B) and the formation of NPs was confirmed. The zeta potential value of the *L. officinalis* extract-based ZnO NPs was –37.7 mV (Figure 2C). According to the dynamic light scattering (DLS) analysis, the hydrodynamic diameter of NPs ranges from 15 nm to 110 nm (Figure 2D). The FT-IR peaks of ZnO NPs were determined at 1 462, 1 402, 1 118, 1 031, 864, 841, 619, and 581 cm^–1^ (Figure 2E). The peaks were observed by XRD analysis as 2θ = 31.7^o^, 34.4^o^, 36.23^o^, 47.2^o^, 56.3^o^, 62.9^o^, 67.9^o^ and 69.1^o^ (Figure 2F).

### Biochemical indicators

**Figure 3:**
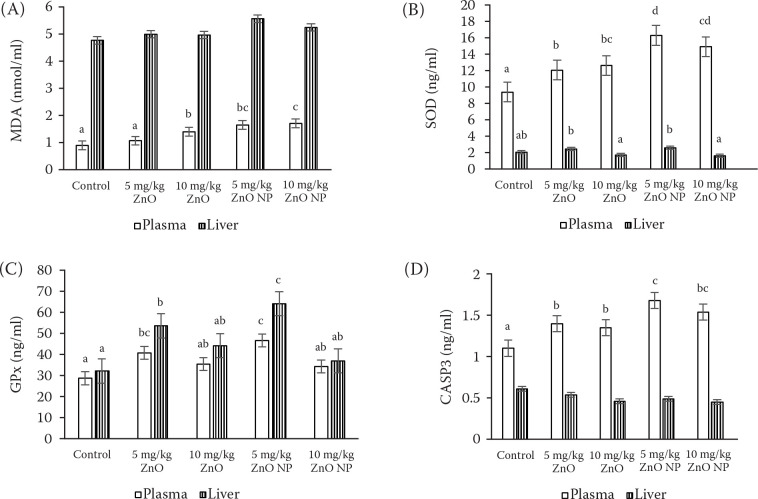
Effect of the ZnO NPs on the plasma and liver MDA levels (A), SOD (B), GPx (C) and CASP3 (D) activities ^a–d^Values in each column with different superscripts are significantly different

A significant difference was not observed between the control and the 5 mg/kg ZnO groups by the plasma MDA concentrations. However, the plasma MDA concentrations were significantly higher in the 10 mg/kg ZnO group compared to the other groups (*P* < 0.001; Figure 3A). Also, no significant differences in the tissue MDA concentrations were determined between the groups (*P* > 0.05; Figure 3A).

The plasma SOD activities were significantly higher in the ZnO and ZnO NPs groups as a control.

Significantly elevated plasma SOD activities were observed in the ZnO and ZnO NP groups relative to the control (*P* < 0.001; Figure 3B). In terms of the tissue SOD activities, the increases were significant in the 5 mg/kg ZnO and ZnO NPs groups compared to the 10 mg/kg ZnO and ZnO NPs groups (*P* < 0.05; Figure 3B). Considering the GPx activities in both the plasma (*P* < 0.05) and tissue (*P* < 0.01), the GPx activities in the treatment groups were higher than the control group. Still, statistically significant increases were detected in the 5 mg/kg ZnO and ZnO NPs groups (Figure 3C).

The plasma CASP3 activities were higher in the treatment groups. These increases were observed to be more prominent, especially in the nanoparticle-applied groups (*P* < 0.01; Figure 3D). No differences between all the groups were observed in the tissue CASP3 activities (*P* > 0.05; Figure 3D).

**Figure 4:**
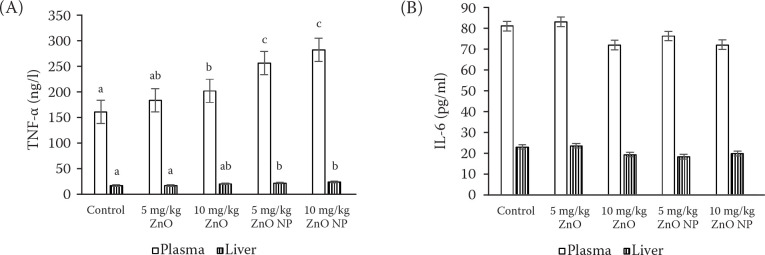
Effect of the ZnO NPs on the plasma and liver TNF-α (A) and IL-6 levels (B) ^a,b^Values in each column with different superscripts are significantly different

The plasma (*P* < 0.001)/tissue (*P* < 0.01) TNF-α concentrations were increased in Zn groups (Figure 4A), with the most significant increase observed in the groups treated with nanoparticles.

**Figure 5:**
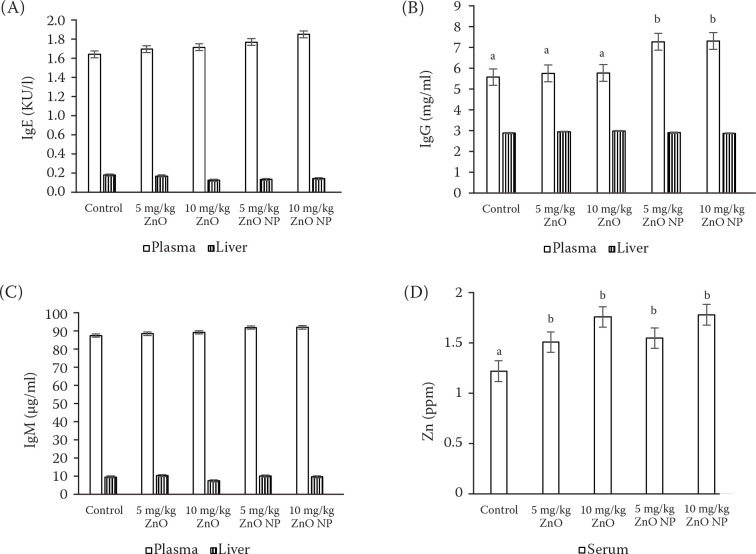
Plasma and liver IgE levels (A), IgG levels (B), IgM levels (C) and serum Zn levels (D) on the effect of the ZnO NPs ^a,b^Values in each column with different superscripts are significantly different

The plasma IgG concentrations were elevated significantly in the ZnO NPs groups (*P* < 0.01; Figure 5B). The applications did not affect the tissue IgG concentrations (*P* > 0.05; Figure 5B). It was determined that both the plasma and tissue IL-6 (Figure 4B), IgE (Figure 5A) and IgM (Figure 5C) concentrations were not affected by the 5 and 10 mg/kg ZnO NPs (*P* > 0.05). Although zinc levels were higher in the groups receiving zinc treatments (*P* < 0.01; Figure 5D), there was no statistical difference among the zinc groups regarding the levels of this element (*P* > 0.05; Figure 5D).

## DISCUSSION

It has been recently reported that the nanoparticle form of minerals has higher bioavailability due to greater catalytic efficiency, surface area and activity, and strong adsorption ability (Xiaoli et al. 2017; Heidai Moghadam et al. 2019), but studies on the effects of the nanoparticle form of Zn mineral are limited (Ahmadi et al. 2013; Behera et al. 2020; Majd et al. 2021a; Majd et al. 2021b; Salim et al. 2024).

According to the SEM images, ZnO NPs (mean diameter 33 nm, spherical) were synthesised by using a *Lavandula officinalis* extract. Abbasi-Oshaghi et al. (2018) reported that ZnO NPs with spherical and polygonal morphology synthesised by the solvothermal method have a diameter between 20–30 nm and tend to agglomerate. Akintunde et al. (2021) synthesised ZnO NPs by taking advantage of both the reducing and capping properties of a *Moringa oleifera* leaf extract. Researchers noted that biosynthesised ZnO NPs were distributed in the 10–25 nm range, and nanorods had hexagonal morphology. Majd et al. (2021a) and Majd et al. (2021b) stated that ZnO NPs, synthesised with an *Aloe vera* extract, were distributed between 20–50 nm. According to the data, the morphology and size of the NPs depend on the concentration and species of the plant extract. Metallic NPs have surface plasmon resonance (SPR) absorption due to the free electrons in the transmission bands. The SPR absorption points of ZnO NPs synthesised by the solvothermal method were determined at 365 nm (Abbasi-Oshaghi et al. 2018). The characteristic SPR absorption bands of ZnO NPs synthesised by a *Poncirus trifoliata* extract were identified at 327 nm (Nagajyothi et al. 2013). There is also a study reporting that the UV absorbance values of ZnO NPs vary between 358–375 nm depending on the concentration of the *Aloe vera* extract (Sangeetha et al. 2011). According to the UV analysis, a red shift was observed at the optical absorption points as a result of electron excitation from the valence to the conduction band. With the increase in the NP size, a shift towards long wavelengths (red shift) is observed in the optical absorption spectra, and a blue shift is observed with the decrease in the size of the nanoparticles (Awad et al. 2024). However, the high DLS sizes of ZnO NPs compared to the SEM analysis is due to the coating property of the plant extract used in biological synthesis (Koca and Duman 2019). According to the literature, factors such as the synthesis method, metal salt and plant concentration, and plant species directly affect the morphology (such as the shape, SPR absorption, size, and distribution) and other structural properties of ZnO NPs (Sangeetha et al. 2011; Nagajyothi et al. 2013; Abbasi-Oshaghi et al. 2018; Akintunde et al. 2021; Majd et al. 2021a; Majd et al. 2021b).

The negatively low zeta potential causes push forces between the particles. Thus, it prevents the clustering of NPs and ensures that particles remain more stable. NPs, zeta surface charge lower than –25 mV, have high stability (Gorbe et al. 2016). In the presented study, according to SEM images, ZnO NPs (average diameter 33 nm, spherical) synthesised using an *L.* *officinalis* extract were found to be quite stable. Functional groups that play a role in the synthesis of ZnO NPs have been determined by FT-IR analysis. In the FT-IR diagram, the peaks observed at 1 462, 1 402, 1 118 and 1 031 cm^–1^ indicate the presence of alkane (C-H), alcohol (O-H), aliphatic ether (C-O) and amine (C-N) groups, respectively. The 864 and 841 cm^–1^ peaks in the diagram are associated with aromatic compounds (C-H). The presence of the metal-oxide (Zn-O) complex was recorded, with peaks observed at 619 and 581 cm^–1^. The crystallinity of ZnO NPs was detailed by XRD analysis. The main diffractions with 2θ values of 31.7^o^, 34.4^o^, 36.23^o^, 47.2^o^, 56.3^o^, 62.9^o^, 67.9^o^ and 69.1^o^ correspond to (1 0 0), (0 0 2), (1 0 1), (0 1 2), (1 1 0), (1 0 3), (1 1 2) and (2 0 1) crystal planes, respectively (JCPDS Card No. 36-1451). This result is consistent with previous studies (Doan Thi et al. 2020; Dogaroglu et al. 2023). The peaks of the XRD indicate the crystalline and hexagonal phases of ZnO NPs.

It is reported that the increase in the surface area of nanomaterials can lead to toxicity when they are reactive and can cause oxidative stress in the organism, enter the circulation, accumulate in the target tissues and organs in the body and cause damage. It has been observed that as the concentration of ZnO NPs increases, the cellular damage also increases, and it has been reported that it causes a significant increase in the levels of MDA, a lipid peroxidation indicator, in the serum, heart, liver and kidney, and decreases in the activities of SOD, GPx and CAT, which are antioxidant parameters; it has been stated that it has harmful effects on the body at doses of 10, 25, 50, 100, 200, 300 and 600 mg/kg, except for 5 mg/kg, which has beneficial effects (Amer and Karam 2018; Ansar et al. 2018; Heidai-Moghadam et al. 2019; Rani et al. 2018; Abdel-Magied and Shedid 2020; Moatamed et al. 2019; Aboulhoda et al. 2020; Ekhlasian et al. 2023; Bautista-Perez et al. 2024; Noorin et al. 2024; Mirzaei et al. 2025).

Mirzaei et al. (2025) investigated the effects of 5 and 10 mg/kg ZnO NPs on the oxidative stress, apoptosis pathways and inflammatory, CASP3 activities, nitric oxide concentrations, antioxidant capacity and the activity of various biochemical factors, and revealed that antioxidant enzyme gene expression and activity were significantly increased, while in rats the apoptosis and inflammation pathways were significantly reduced by 5 mg/kg ZnO NPs. The researchers found that treating the animals with 5 mg/ZnO NPs revealed potential hepatoprotective properties, while ZnO NPs at doses above 10 mg/kg showed toxic effects. There are also other studies showing that doses of ZnO NPs above 5 mg/kg have harmful effects on Wistar Albino rats (Amer and Karam 2018; Ansar et al. 2018; Heidai-Moghadam et al. 2019; Rani et al. 2018; Abdel-Magied and Shedid 2020; Moatamed et al. 2019; Aboulhoda et al. 2020; Akintunde et al. 2021; Ekhlasian et al. 2023; Bautista-Perez et al. 2024; Noorin et al. 2024). In one study (Amer and Karam 2018), it was found that when 5.6 mg/kg ZnO NP was administered to rats (i.p. for 28 days, 3 days a week), the brain MDA levels increased, whereas the total antioxidant capacity (TAC) and GPx activity were significantly decreased. In another study (Akintunde et al. 2021), Wistar Albino rats administered 10 mg/kg of ZnO nanoparticles synthesised through green methods in olive oil for 10 days showed a numerical increase in the brain MDA concentrations and a significant decrease in the SOD activity. However, the administration of 10 mg/kg ZnO nanoparticles in commercial form to rats has also been associated with non-significant increases in the heart MDA, SOD, and GPx (Abdel-Magied and Shedid 2020). It has been reported that the oral administration of 10 mg/kg ZnO NP every three days for 3 months in Wistar Albino rats caused damage in the heart, liver, kidney, and brain (Bautista-Perez et al. 2024); in addition, higher doses (25, 50, 100, 200, 300, and 600 mg/kg; i.p.) caused a significant increase of MDA, a lipid peroxidation product, in the serum, heart, liver, and kidney, and decreased the antioxidant indicators SOD, GPx, and CAT activities (Amer and Karam 2018; Ansar et al. 2018; Heidai-Moghadam et al. 2019; Rani et al. 2018; Abdel-Magied and Shedid 2020; Moatamed et al. 2019; Aboulhoda et al. 2020; Ekhlasian et al. 2023; Noorin et al. 2024).

In the present study, the administration of 5 and 10 mg/kg (body weight/every other day; i.p.) ZnO NP to rats increased the plasma MDA concentrations, an indicator of lipid peroxidation, and the SOD activity, one of the antioxidant enzymes. However, the liver and plasma GPx activity showed a significant increase with the 5 mg/kg dose of ZnO and ZnO NPs, while it did not show any change with the 10 mg/kg dose. In general, it is thought that the increases seen in antioxidant indicators are in order to compensate for the increase in the MDA levels, which is an oxidative indicator, and thus to ensure the oxidant-antioxidant balance. In this study, the lower increase in GPx activity observed in the 10 mg/kg ZnO NP group may be attributed not only to the toxicity and oxidative stress caused by the increased surface area of the nanomaterial when reactive, but also to the possibility that the pro-oxidant effects resulting from high-dose ZnO and ZnO NP exposure could not be adequately counterbalanced by the antioxidant defence mechanisms.

Oxidative stress is one of the factors that can lead to the formation of apoptotic cells (D’Arcy 2019). It has been stated that in cases such as oxidative stress and DNA damage occurring in metabolism, active CASP9 occurring in the intrinsic pathway and CASP8 activated in the extrinsic pathway both activate CASP3 and then CASP6 and 7, respectively (Elmore 2007). In studies where ZnO NPs were applied at different doses (200, 600 mg/kg, 1 g/kg, oral, commercial form), the heart CASP3 activities of Wistar Albino rats were examined and a significant increase was found in this parameter (Abdel Baky et al. 2013; Ekhlasian et al. 2023). In another study (Al-Rasheed et al. 2014), when 600 mg/kg and 1 g/kg ZnO NP (commercial form) were administered orally to Wistar Albino rats, the liver CASP3 activities increased at both doses. It was also determined that the CASP3 activities increased immunohistochemically with the increasing ZnO NP dose (1.4, 1.75, 7.1 and 8.9 mg/kg, i.p.) and duration (1, 7 and 21 days) in Wistar Albino rats (Sizova et al. 2019). Additionally, it has been reported that different doses of ZnO NPs (100, 200, 300 mg/kg/day) increased the CASP3 mRNA expression in Sprague-Dawley rats (Aboulhoda et al. 2020). Consistent with the findings of previous studies (Abdel Baky et al. 2013; Al-Rasheed et al. 2014; Aboulhoda et al. 2020; Ekhlasian et al. 2023), ZnO NPs also caused an increase in the plasma CASP3 in this study, suggesting that this increase may be related to the possible induction of cell damage, which is thought to develop due to oxidative stress.

Cytokines are polypeptides produced and secreted by various cell types and play a role in biological events such as the control of cell division and differentiation, healing of wounds, regulation of immune and inflammatory mechanisms, haematopoiesis, bone formation and alteration of cell metabolism (Oppenheim 2001; Zhang and An 2007). Cytokines are also immune defence reactions produced during infection or damage in the body by releasing signal molecules between immune cells (Abdel-Magied and Shedid 2020). Various studies have shown that the increase in oxidative stress parameters, which negatively affects the antioxidant defence mechanism for different reasons, is accompanied by increased cytokines (Abdel Baky et al. 2013; Abdel-Magied and Shedid 2020). Some *in vitro* studies have shown that nanoparticles can stimulate macrophages through reactive oxygen species (ROS) and calcium signalling pathways and the production of cytokines such as TNF-α and IL-6 (Borm et al. 2006; Sheweita and Khoshhal 2007). Amer and Karam (2018) found a significant increase in the brain TNF-α and IL-6 concentrations in the study in which they administered 5.6 mg/kg ZnO NPs via the i.p. route 3 days a week for 28 days. Sizova et al. (2019) said that the serum TNF-α concentrations were elevated significantly in Wistar Albino rats with the administration of several doses of ZnO NP (1.4, 1.75, 7.1 and 8.9 mg/kg) at various times (days 1, 7, and 21). The group administered with 8.9 mg/kg ZnO NP showed a decrease on the 7^th^ day compared to the 1^st^ day, and an increase was observed on the 21^st^ day in all the groups given 1.4, 1.75 and 8.9 mg/kg ZnO NP, and also no difference was found between the IL-1 levels.

In Wistar Albino rats, it was reported that 10 mg/kg ZnO NPs (i.p.) did not affect the TNF-α and IL-18 concentrations (Abdel-Magied and Shedid 2020). In previous studies at a level of 600 mg/kg ZnO NPs (commercial form) (Faddah et al. 2012; Abdel Baky et al. 2013; Al-Rasheed et al. 2014; Abdelkarem et al. 2016) and a level of 1 g/kg (Abdel Baky et al. 2013; Al-Rasheed et al. 2014; Faddah et al. 2012) were applied orally to Wistar Albino rats (Abdel Baky et al. 2013; Al-Rasheed et al. 2014; Abdelkarem et al. 2016; Faddah et al. 2012), it was revealed that the serum TNF-α and IL-6 concentrations of the animals increased in both doses. In this study, as in agreement with the findings of some authors (Abdel Baky et al. 2013; Abdelkarem et al. 2016; Amer and Karam 2018; Sizova et al. 2019; Faddah et al. 2012), increases were observed in the serum/plasma TNF-α and IL-6 and liver TNF-α concentrations as supported by an increase in the oxidative stress. Compared to the ZnO groups, the increase in apoptotic marker CASP3 activity and cytokine TNF-α levels in the 5 and 10 mg/kg ZnO NP groups also supports the increase in the MDA levels. These effects are thought to be due to oxidative stress (Ansar et al. 2018; Heidai-Moghadam et al. 2019; Rani et al. 2018; Abdel-Magied and Shedid 2020; Moatamed et al. 2019; Aboulhoda et al. 2020) caused by the long-term retention in the circulatory system and causing toxicity due to the small size, large catalytic efficiency, surface area, activity and effective adsorption ability of the nanoparticle form of Zn (Fard et al. 2015; Xiaoli et al. 2017; Heidai-Moghadam et al. 2019).

Immunoglobulins, one of the most distinctive features of immunity, are biomolecules in glycoprotein structure that contribute to immunity against pathological threats by binding antigens and forming the antigen-antibody complex. Immunoglobulin G, the most abundant in blood, lymph, peritoneum, and cerebrospinal fluid, binds antigens and eliminates their harmful effects (Sun et al. 2020; Keyt et al. 2020). The increase in circulating antibodies is believed to result from various inflammatory cytokines, including TNF-α, which may influence the immunoglobulin production during inflammatory responses (Pujalte et al. 2011). In studies on Wistar Albino rats treated with 600 mg/kg (Faddah et al. 2012; Al-Rasheed et al. 2014; Abdelkarem et al. 2016) and 1 g/kg (Faddah et al. 2012; Al-Rasheed et al. 2014) ZnO NPs (in commercial form) via the oral route, it was revealed that the serum IgG concentrations were elevated in these doses as a result of the zinc oxide nanoparticle administration is thought to be an immune response caused by the toxicity (Xiaoli et al. 2017; Heidai-Moghadam et al. 2019) of the nanoparticles. Similarly, in the presented study, it can be explained that the increases in the plasma IgG antibodies that may affect the immunoglobulin production during inflammatory responses in the ZnO NP groups may be due to the increased TNF-α inflammatory cytokines (Pujalte et al. 2011), thus the metabolism may develop a strong defence mechanism against oxidative stress.

Using nanoparticles of the same metal increases the metal pool within the organism (Cuillel et al. 2014); that is, increases in the liver, kidneys, intestine, and plasma concentrations were reported (Park et al. 2014). In a study conducted by Abdel-Magied and Shedid (2020), in Wistar Albino rats, no difference in the heart Zn levels was observed between the control and the group treated with 10 mg/kg ZnO NPs. However, the 300 mg/kg ZnO NPs group noted a statistically significant increase. In another study conducted on Wistar Albino rats injected with 1.4, 1.75, 7.1 and 8.9 mg/kg ZnO NPs intraperitoneally, the liver Zn levels increased significantly with the 1.75 and 8.9 mg/kg ZnO NPs (Sizova et al. 2019). Wang et al. (2016) who fed mice with 50, 500, and 5 000 mg/kg ZnO NPs supplemented diets for a long time, also reported that 5 000 mg/kg ZnO NPs resulted in a significant increase in the liver Zn concentration. In this presented study, as revealed in previous studies (Wang et al. 2016; Sizova et al. 2019; Abdel-Magied and Shedid 2020), it was observed that the serum Zn concentrations were higher in the Zn groups. Although a numerical increase was determined in parallel with the increasing Zn concentrations, this increase was not found to be statistically significant. It suggests that these changes in the serum Zn concentrations may be due to different doses of ZnO NPs.

In this study, the 5 and 10 mg/kg (body weight; every other day; i.p.) ZnO NP administration to rats increased the plasma MDA, CASP3, plasma and liver TNF-α concentrations of oxidative stress indicators and SOD, GPx activities of antioxidant enzymes and IgG concentrations. Although the increases observed in the antioxidant indicators with the low dose (5 mg/kg) ZnO NP administration are thought to represent a compensatory response aimed at balancing the elevated oxidant indicators and thus maintaining the oxidant-antioxidant balance, antioxidant defence systems may have been overloaded or suppressed as a result of the higher dose (10 mg/kg) ZnO NP administration. This situation can be explained by the inhibition of enzyme systems or a deterioration in protein structure due to oxidative damage with the progression of oxidative stress. It should also be considered that high-dose nanoparticles may increase the cellular damage by causing toxic accumulation in biological systems.

As a result, it can be said that the effectiveness of nanoparticles may vary depending on the synthesis method, concentration, and application time. To understand the effects of ZnO NPs on oxidative stress, studies using different durations and doses will be useful.
